# Spatial Structure Formation by RsmE-Regulated Extracellular Secretions in Pseudomonas fluorescens Pf0-1

**DOI:** 10.1128/jb.00285-22

**Published:** 2022-09-27

**Authors:** Anton F. Evans, Meghan K. Wells, Jordan Denk, William Mazza, Raziel Santos, Amber Delprince, Wook Kim

**Affiliations:** a Department of Biological Sciences, Duquesne University, Pittsburgh, Pennsylvania, USA; NCBI, NLM, National Institutes of Health

**Keywords:** biosurfactants, cell-cell interaction, extracellular matrix, mutational studies, variable phenotypes

## Abstract

Cells in microbial communities on surfaces live and divide in close proximity, which greatly enhances the potential for social interactions. Spatiogenetic structures are manifested through competitive and cooperative interactions among the same and different genotypes within a shared space, and extracellular secretions appear to function dynamically at the forefront. A previous experimental evolution study utilizing Pseudomonas fluorescens Pf0-1 colonies demonstrated that diverse mutations in the *rsmE* gene were repeatedly and exclusively selected through the formation of a dominant spatial structure. RsmE’s primary molecular function is translation repression, and its homologs regulate various social and virulence phenotypes. Pseudomonas spp. possess multiple paralogs of Rsm proteins, and RsmA, RsmE, and RsmI are the most prevalent. Here, we demonstrate that the production of a mucoid polymer and a biosurfactant are exclusively regulated through RsmE, contradicting the generalized notion of functional redundancy among the Rsm paralogs. Furthermore, we identified the biosurfactant as the cyclic lipopeptide gacamide A. Competition and microscopy analyses showed that the mucoid polymer is solely responsible for creating a space of low cellular density, which is shared exclusively by the same genotype. Gacamide A and other RsmE-regulated products appear to establish a physical boundary that prevents the encroachment of the competing genotype into the newly created space. Although cyclic lipopeptides and other biosurfactants are best known for their antimicrobial properties and reducing surface tension to promote the spreading of cells on various surfaces, they also appear to help define spatial structure formation within a dense community.

**IMPORTANCE** In densely populated colonies of the bacterium Pseudomonas fluorescens Pf0-1, diverse mutations in the *rsmE* gene are naturally selected by solving the problem of overcrowding. Here, we show that RsmE-regulated secretions function together to create and protect space of low cell density. A biosurfactant generally promotes the spreading of bacterial cells on abiotic surfaces; however, it appears to function atypically within a crowded population by physically defining genotypic boundaries. Another significant finding is that these secretions are not regulated by RsmE’s paralogs that share high sequence similarity. The experimental pipeline described in this study is highly tractable and should facilitate future studies to explore additional RsmE-regulated products and address why RsmE is functionally unique from its paralogs.

## INTRODUCTION

Central to the architecture of microbial communities is the extracellular matrix (1–8), a dynamic cumulus of compounds produced by individual cells that physically define both the spatial arrangements within the community and the three-dimensional boundaries. Micrometer-scale spatiogenetic structures readily emerge within surface-grown communities as individual cells produce identical copies of themselves in a given area (9–11). Competition between different genotypes leads to the spatial enrichment of a particular genotype, producing macroscopic regions that stem from a recent common ancestor (10, 12–15). Individual phenotypes can positively or negatively impact the fitness of neighboring cells, including the consumption of limiting nutrients and the secretion of enzymes and toxins that promote or discourage the growth of neighboring cells (8, 16–18). Mechanistic understanding of how individual phenotypes antagonize or synergize with another clearly carries both fundamental and clinical significance.

Researchers employ various experimental approaches to study the interactive dynamics of microbial cells within a community, whether the approaches be computational (9, 10, 19) or empirical on a variety of abiotic surfaces (5, 20–22). We previously described a model system based on Pseudomonas fluorescens colonies which showed how spatial structures rapidly evolved within clonal aggregates (23). Mucoid patches repeatedly emerge on the surface of aging colonies due to the activities of specific mutants, where they expand space and decrease local density. Remarkably, a mutation in a single gene, *rsmE*, was responsible for each and every case of over 500 independently derived mucoid patches. Importantly, *rsmE* mutants shared the same growth rate in isolation as the parent cells, and the evolutionary advantage specifically required the proximal presence of the parent cells. These observations collectively suggest that RsmE-regulated phenotypes physically act to create dominant spatial structures in a densely populated bacterial colony.

RsmE belongs to the CsrA/Rsm family, and its homologs are regulators of social and virulence phenotypes in *Gammaproteobacteria* (24, 25). CsrA was the first member of the family to be discovered 3 decades ago in Escherichia coli (26), and its homologs are now known to be present in over 2,900 species (27). CsrA/Rsm proteins interact with diverse mRNA (25, 28–30) and primarily function as a translation repressor by either directly or indirectly blocking their respective Shine-Dalgarno sequence (31–34). CsrA also possesses additional regulatory functions that impact Rho-dependent transcription attenuation, mRNA stabilization and destabilization, and even activation of translation (32). In contrast to CsrA in *Enterobacteriaceae*, Pseudomonas spp. possess varied numbers of Rsm paralogs (27). Rsm paralogs were initially characterized to repress the production of secondary metabolites and are generally described to overlap or cumulate in function (35–41).

Although the three paralogs in P. fluorescens (RsmE, RsmA, and RsmI) share high sequence similarity, the exclusive selection of mutations in the *rsmE* locus (23) suggests functional specificity of RsmE from its paralogs. Here, we show that all three Rsm paralogs are expressed, but RsmE uniquely governs the production of both a mucoid polymer and a biosurfactant. The biosynthetic genes of the mucoid polymer were previously described (42), and we identified the biosynthetic genes of the biosurfactant in this study. Competition and microscopy analyses of the extracellular polysaccharide and biosurfactant mutants revealed that these extracellular secretions function collectively to confer a fitness benefit as a direct result of the spatial structures they form.

## RESULTS

### RsmE, RsmA, and RsmI in P. fluorescens Pf0-1 are highly conserved in sequence and all three respective genes are expressed.

**FIG 1:**
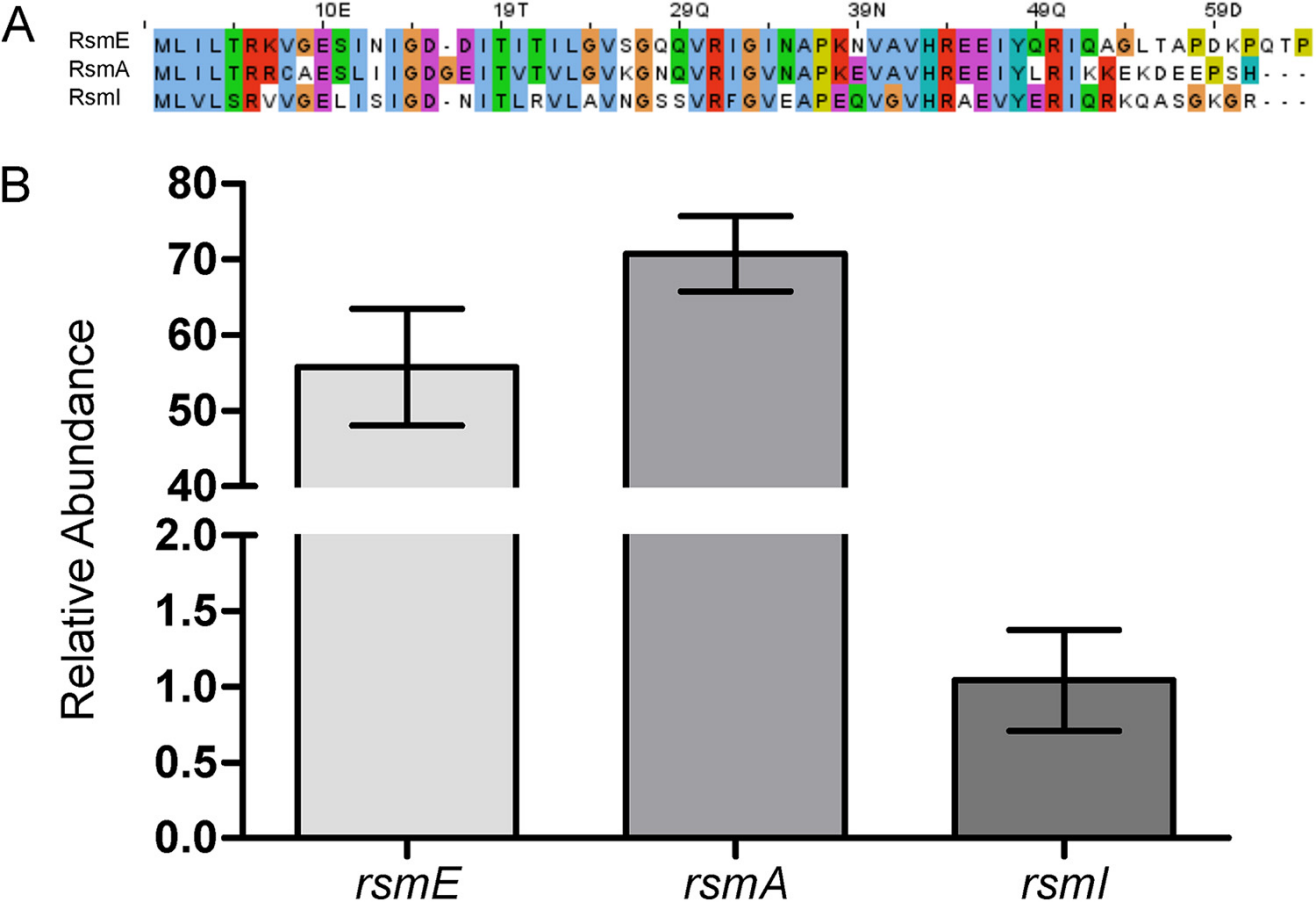
Rsm paralogs in P. fluorescens Pf0-1 share a highly conserved sequence, and their respective genes are simultaneously expressed. (A) Sequence alignments of Rsm paralogs in Pseudomonas fluorescens Pf0-1 with ClustalX show similarities of amino acid sequences and chemical properties (65). (B) Expression of *rsmE*, *rsmA*, and *rsmI* genes assessed in WT by qPCR. Transcripts of all three genes were detected; shown here is the relative abundance of each transcript using the 2^−ΔΔ^*^CT^* method, with comparison to that of the least abundantly expressed, *rsmI*. Plotted are the means of three biological replicates with three technical replicates for each biological replicate, and the error bars represent the standard deviations of the means.

P. fluorescens Pf0-1 possesses three Rsm paralogs, RsmA, RsmE, and RsmI, which share high sequence similarity (Fig. 1A). We sought to first determine whether or not the three corresponding genes are expressed. Quantitative PCR confirmed that all three genes are indeed expressed at the time of sampling (Fig. 1B). We extracted mRNA from wild-type (WT) cells growing in colonies after 3 days of incubation, which coincided with the timing of the natural emergence of *rsmE* mutants as visible mucoid patches (23). These results showed that the exclusive selection of *rsmE* mutations in our previous experimental evolution study (23) was not simply due to the absence of *rsmA* and *rsmI* expression under the same experimental conditions.

### RsmE specifically regulates the production of a mucoid polymer and biosurfactant.

**FIG 2:**
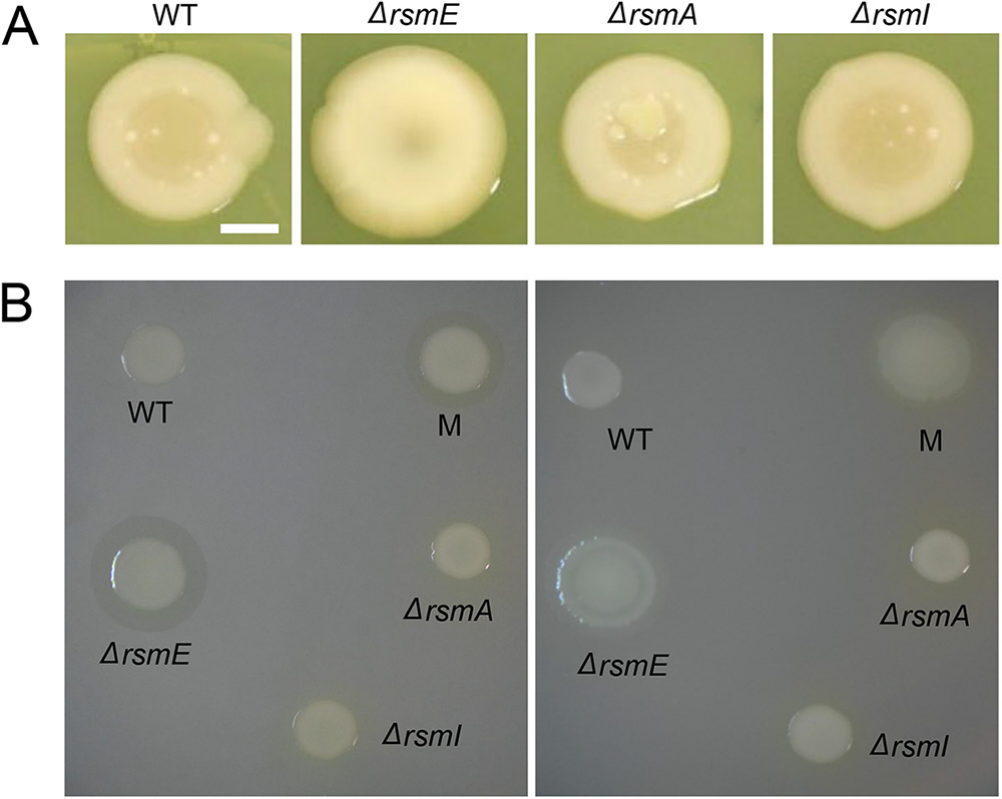
Both the mucoid polymer and the biosurfactant are regulated by RsmE, but not by RsmA or RsmI. (A) Colony morphology comparisons of WT and deletion mutants of *rsmE*, *rsmA*, and *rsmI*. Liquid cultures were spotted on PAF plates 7 days prior to capturing the images. Only the *ΔrsmE* strain was mucoid in appearance, and new mucoid patches naturally emerged in WT, Δ*rsmA*, and Δ*rsmI* colonies that characteristically represented *de novo rsmE* mutations. Scale bar, 10 mm. (B) Comparison of biosurfactant production on the dull (left) and shiny (right) sides of the polycarbonate membrane overlaid on PAF. The M strain is a naturally selected mutant from a WT colony and harbors a frameshift mutation in *rsmE*. Only the Δ*rsmE* and M strains produced the biosurfactant ring on the dull side, which promoted spreading of cells on the shiny side of the membrane.

Experimentally selected *rsmE* mutants visibly produce a mucoid polymer and/or a biosurfactant (23), which suggests that specific mutations differentially impact RsmE’s functions. To determine if these extracellular secretions are commonly regulated by the three Rsm homologs, we constructed deletion mutants for the three genes. Comparison of colony morphologies showed that only the *rsmE* mutant exhibited mucoidy (Fig. 2A). In addition, mucoid patches consistently emerged in colonies of the WT, *rsmA* mutant, and *rsmI* mutant (Fig. 2A); these colonies were characteristic of naturally mutated *rsmE* (23). These results confirmed that the production of the mucoid polymer is specifically regulated by RsmE. We next compared biosurfactant production on a polycarbonate membrane overlaid on the agar surface. Production of the biosurfactant on the shiny side of the membrane allows the colony to spread out radially, but the cells remained trapped on the dull side of the membrane while the biosurfactant spreads out unhindered (23). Only the *rsmE* mutant produced a visible ring on the dull side of the membrane and also spread out on the shiny side of the membrane (Fig. 2B). Furthermore, genetic complementation of the *rsmE* knockout strain with the native *rsmE* locus restored the WT phenotype (see Fig. S1 in the supplemental material). These results confirmed that the production of both the mucoid polymer and the biosurfactant is uniquely governed by RsmE from its paralogs.

### Identification of the biosurfactant gacamide A.

**FIG 3:**
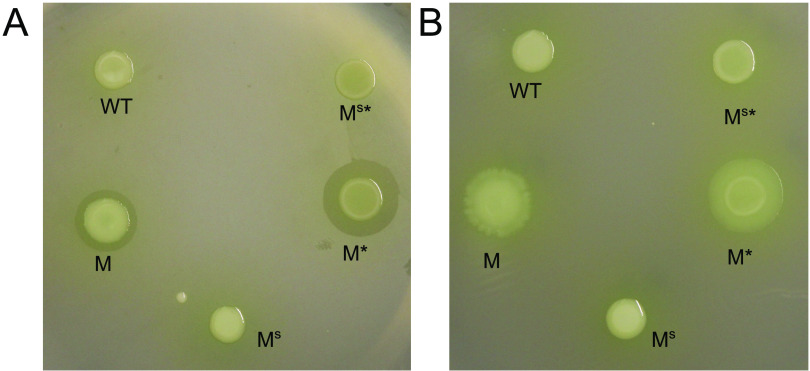
Deletion of the Pfl01_2211 locus abolished biosurfactant production. Shown are the results from the dull side (A) and the shiny side (B) of the polycarbonate membrane. M (*rsmE* mutant) and M* (M with the mucoid polymer biosynthesis gene [Pfl01_3834] deleted) produced the biosurfactant and spread on the surface, but M^S^ (M with Pfl01_2211 deleted) and M^S^* (M with both Pfl01_3834 and Pfl01_2211 deleted) failed to do so, as did the WT with an unaltered *rsmE* gene. These results confirmed that the Pfl01_2211-2213 cluster encodes the biosynthetic genes of the biosurfactant, which is now known to be gacamide A.

The biosynthetic genes of the mucoid polymer were previously characterized as encoding a glucose-rich extracellular polysaccharide, and a corresponding gene was deleted in a mucoid (M) strain with a frameshift mutation in *rsmE* (23) to produce the nonmucoid M* strain (42). To identify the biosynthetic genes of the biosurfactant, we carried out random transposon mutagenesis in the M* strain background. Seven mutants were independently isolated that no longer produced the secretion on the dull side of the polycarbonate membrane and failed to spread out on the shiny side of the membrane. All transposon insertion sites were mapped to three contiguous loci (annotated as Pfl01_2211, Pfl01_2212, and Pfl-1_2213), which were recently demonstrated to encode nonribosomal peptide synthetases (43) that produce the cyclic lipopeptide gacamide A (44). Cyclic lipopeptides are indeed classified as surfactants, and they contribute to surface motility and biofilm formation in many Pseudomonas spp. (45, 46). Given that four independent transposon insertions occurred in the Pfl01_2211 locus, we constructed a corresponding in-frame deletion mutant in the M strain to produce the M^S^ strain, and the same mutation was also introduced in the nonmucoid M* strain to produce the M^S^* strain. Neither M^S^ nor M^S^* produced the biosurfactant ring on the dull side of the membrane and the spreading phenotype on the shiny side of the membrane (Fig. 3), confirming that the Pfl01_2211-Pfl-1_2213 cluster encodes the production of the biosurfactant. Importantly, M* maintained the production of the biosurfactant and M^S^ maintained the production of the mucoid polymer (Fig. 3), which showed that the biosynthesis of these two secreted products is not genetically linked but that both are regulated by RsmE.

### Both the mucoid polymer and biosurfactant confer a competitive advantage.

**FIG 4:**
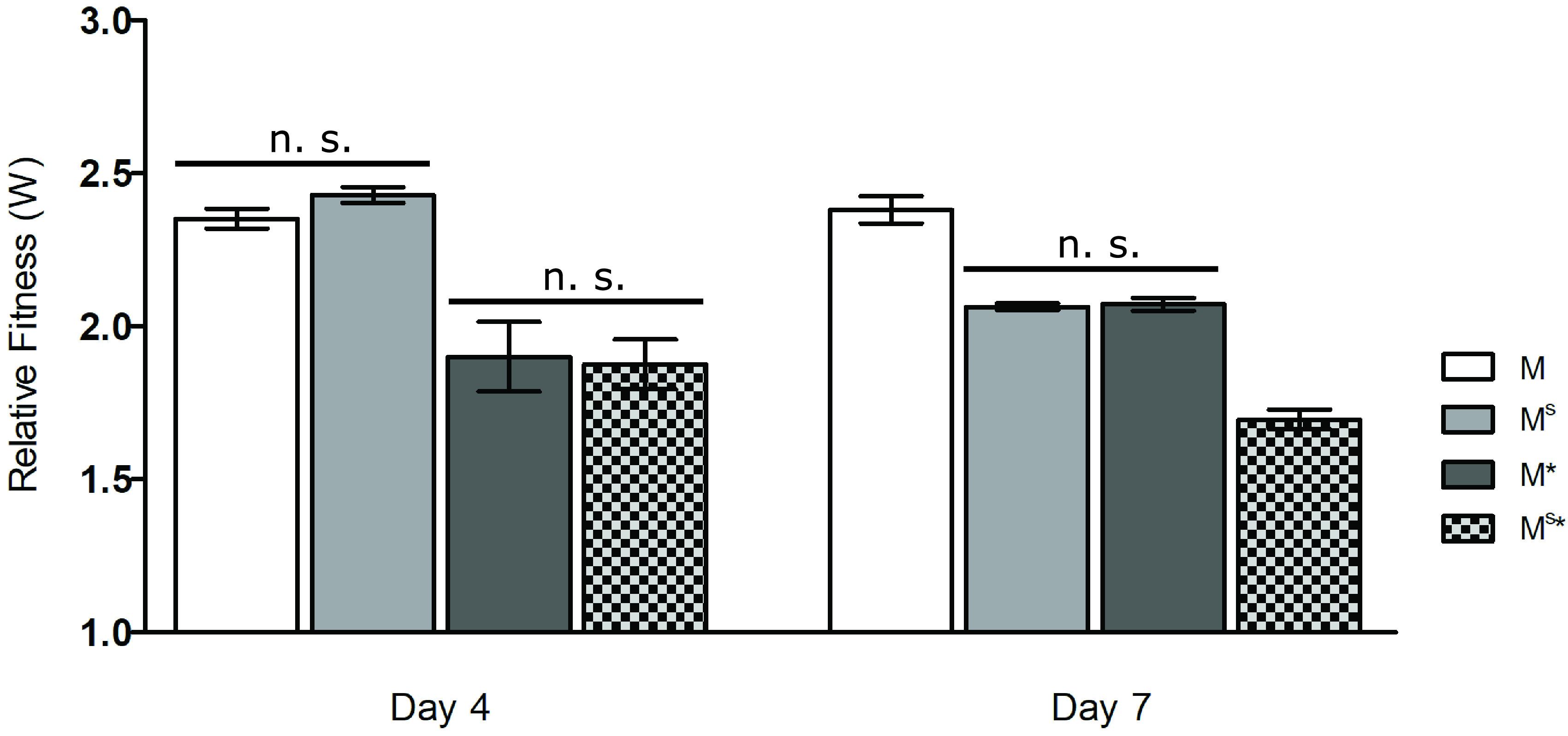
Competitions of M, with or without mucoid polymer and/or biosurfactant production, against WT showed varying levels of relative fitness over time. WT was chromosomally tagged with streptomycin resistance and all mutants were tagged with kanamycin resistance, and these resistance markers produced neutral relative fitness in P. fluorescens Pf0-1 (23). Error bars represent the standard deviations of the mean relative fitness (mutant over WT) calculated from three independent populations after 4 and 7 days of incubation. Data from each time point were analyzed by ANOVA (*P* < 0.0001), and Tukey’s honest significant difference test showed that all pairwise comparisons were significantly different (*P* < 0.05) except for those indicated as nonsignificant (n.s.). A relative fitness (*W*) of 1 indicates equal fitness of the mutant and WT, and a *W* value of >1 indicates that the mutant outcompeted the WT. Both the mucoid polymer and the biosurfactant provided a competitive advantage. However, M^S^* (*rsmE* mutant with biosynthesis genes of both secretions deleted) still outcompeted the WT, suggesting that additional RsmE-regulated products contribute to the competitive advantage of M (*rsmE* mutant) against the WT.

We previously showed that the *rsmE* knockout mutant outcompetes the WT strain in cocultured colonies but not in liquid cocultures, which indicates that RsmE-regulated products provide benefit exclusively in a structured population (23). To assess the contributions of the RsmE-regulated mucoid polymer and the biosurfactant, we independently competed M, M^S^, M*, and M^S^* against the WT in cocultured colonies and assessed their fitness relative to the WT. All four strains outcompeted the WT throughout the duration of the experiments (Fig. 4), with M and M^S^ being nearly equal in fitness and M* and M^S^* exhibiting decreased fitness at day 4. However, we observed reduced fitness in all secretion mutants compared to M by day 7, with M^S^ and M* being comparable and M^S^* exhibiting a further reduction. Such stepwise decreases in fitness indicate that each secreted product independently confers a competitive advantage and the two secretions also likely function in an additive manner. Furthermore, the fact that M^S^* retains the ability to outcompete the WT indicates that there are additional RsmE-regulated genes that contribute to M’s dominance over the WT.

### The mucoid polymer creates space and the biosurfactant prevents diffusion of the mucoid polymer at the colony surface.

The temporal differences in the relative fitness between M^S^ and M* (Fig. 4) suggest that the mucoid polymer plays a more significant role early in the competition. Importantly, M^S^*, the double secretion mutant, exhibited an equal growth profile compared to both the WT and M as monoculture in either liquid or colonies (see Fig. S2). The M data here recapitulate the results from our previous study, which also demonstrated that the competitive advantage of *rsmE* mutants specifically required the formation of spatial structures that decreased local density and provided greater access to oxygen (23).

**FIG 5:**
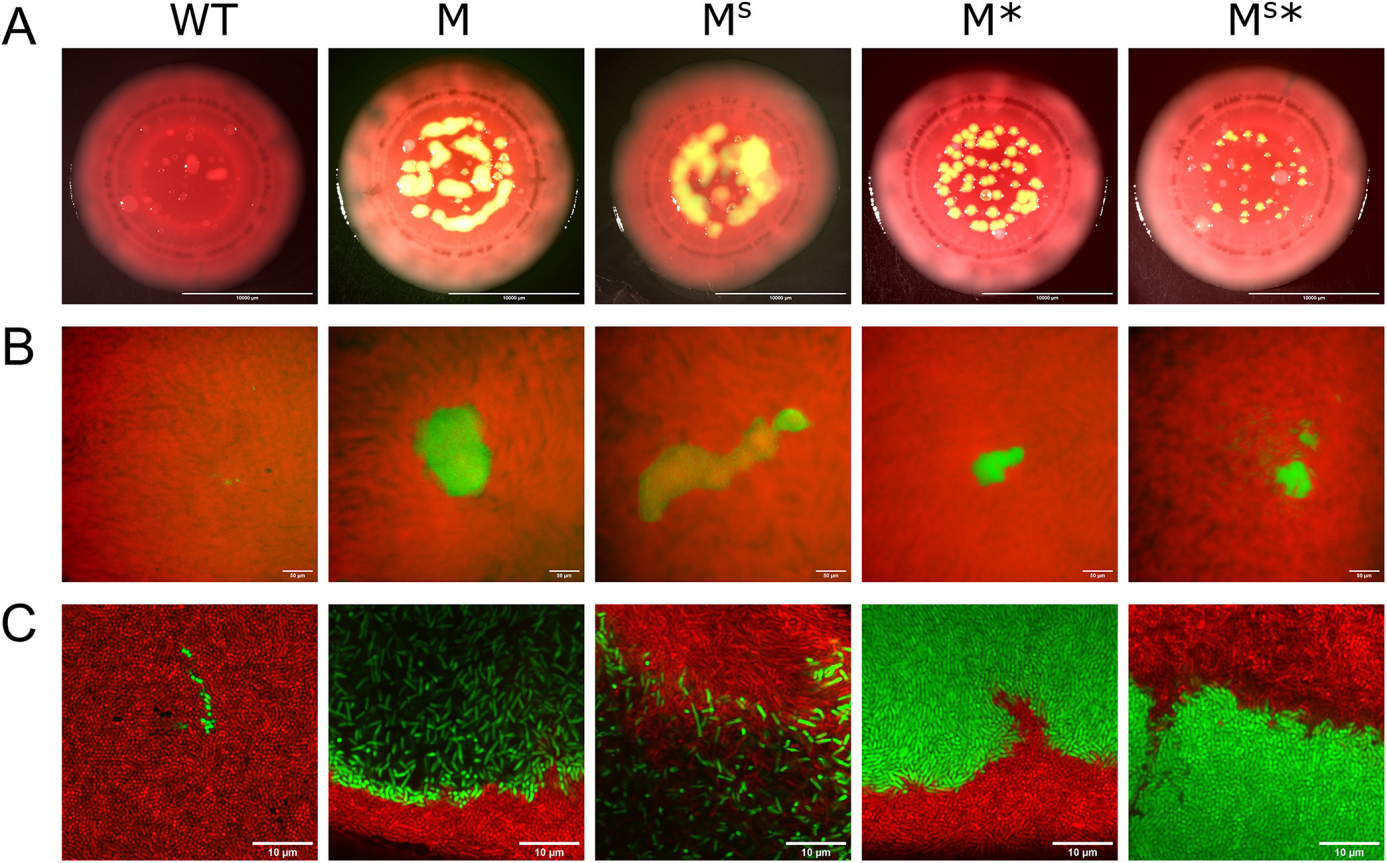
The mucoid polymer and biosurfactant function together in the formation of a dominant spatial structure. Each indicated strain was chromosomally tagged with GFP, heavily underrepresented in a mixture with DsRed Express-tagged WT, and representative cocultured colonies were imaged 5 days later. (A) Epifluorescence microscopy images that captured the entire colony. Scale bar, 10 mm. Each sample showed the natural emergence of red mucoid patches that are characteristic of *de novo rsmE* mutants, stemming from the red fluorescent WT cells. (B) Epifluorescence microscopy images focusing on the surface of individual patches. Scale bar, 50 μm. M^S^* produced unique patches that appeared to be mixed with red WT cells. (See also Fig. S3 in the supplemental material, which shows the green and red channels separated.) (C) Confocal microscopy images at a higher magnification, focused on the boundaries between the mutant and WT. Scale bar, 10 μm. The mucoid polymer is solely responsible for creating the space of low cell density (black space is devoid of cells), and the biosurfactant appears to physically hold the mucoid polymer and producing cells from flowing out from the newly created space. M^S^* produces the smallest patches that are densely filled, as reflected by vertically aligned cells (spheres) similar to the WT:WT spatial organization (left panel). However, M^S^* maintains the ability to form an organized structure that excludes WT cells, suggesting that additional RsmE-regulated products contribute to the spatial dominance of M. See also Fig. S4, in which the green and red channels are separated.

To explore the functional role of the RsmE-regulated mucoid polymer and biosurfactant in spatial structure formation, we carried out epifluorescence and confocal microscopy analyses of our collection of secretion mutants compared to the WT. We first introduced a constitutively expressed *gfp* gene into the chromosome of WT, M, M^S^, M*, and M^S^* strains. Each green fluorescent protein (GFP) strain was mixed with red fluorescence-labeled WT at a respective ratio of 10^−5^:1 to best visualize isolated spatiogenetic structures in colonies after 5 days. Epifluorescence imaging of entire colonies showed isolated green fluorescent patches emerging from mostly red fluorescent WT colonies, with M and M^S^ producing consistently bigger patches than M* and M^S^* (Fig. 5A). Each coculture also produced red fluorescent mucoid patches, which represented *de novo rsmE* mutants naturally emerging from the red fluorescent WT cells (23); however, no green fluorescent patches were observed in the WT:WT colonies. With epifluorescence imaging at a higher magnification, the green fluorescence signal in the smaller patches formed by M* and M^S^* was much more intense than in patches formed by M, and M^S^ patches produced the least intense fluorescence signal (Fig. 5B; see also Fig. S3). Individual patches formed by both M and M^S^ typically merged together with nearby patches through continuous expansion over time, but we consistently observed M^S^ patches to be much more amorphous in structure with less-defined individual boundaries. In contrast, green fluorescent WT patches were rarely observed and appeared to comprise only a few cells.

Confocal imaging using an air-corrected 100× Plan Apo objective provided a clear view of individual green fluorescent cells and their spatial arrangement within a given patch surrounded by red fluorescent WT cells (Fig. 5C; see also Fig. S4). M cells were present at a strikingly lower density compared to the neighboring WT cells, with the characteristic black space that was devoid of cells (23). In addition, M patches were defined by a clear boundary formed with a thin layer of M cells, which appeared to exclude the encroachment of WT cells into the black space. In contrast, M^S^ patches lacked a clear exclusionary boundary, with M^S^ cells appearing to flow over the WT cells. This interpretation was also reflected in the lower-magnification observations of M^S^ patches being more mucoid and amorphous (Fig. 5A) and producing less intense fluorescent signal (Fig. 5B and Fig. S3) compared to patches formed by M. M* and M^S^* both formed much more densely packed patches with clear boundaries against the WT cells, but M^S^* cells appeared to be even more densely packed, as indicated by the uniquely vertical arrangement of cells (Fig. 5C and Fig. S4) and much smaller sizes of individual patches (Fig. 5A). These observations collectively suggested that the mucoid polymer is the primary driver of creating space, while the biosurfactant spatially sequesters the mucoid polymer to prevent their diffusion. However, M^S^* retains the ability to produce a spatiogenetic structure that contrasts greatly from the green fluorescent WT cells, which form small clusters of only a few cells without any organized structure (Fig. 5C and Fig. S4), likely representing daughter cells stemming from initially a single mother cell. As reflected by our relative fitness data (Fig. 4), there appear to be additional RsmE-regulated genes that specifically promote spatial competition in a densely populated colony.

## DISCUSSION

Several members of the *Gammaproteobacteria*, including Pseudomonas spp., possess multiple paralogs of CsrA/Rsm proteins, and their corresponding genes are also present in diverse plasmids and bacteriophages (27). We previously showed that mutations in *rsmE* are exclusively selected as mucoid patches in colonies of P. fluorescens Pf0-1 (23), suggesting that RsmE’s function is not entirely redundant from that of its paralogs, RsmA and RsmI. In this study, we showed that the functions of all three paralogs are accessible to evolutionary selection, and mucoid patches consistently emerge in both *rsmA* and *rsmI* knockout colonies. We have also demonstrated that knocking out *rsmE* results in the production of two visible extracellular secretions, a mucoid polymer and a biosurfactant, but neither is produced in *rsmA* or *rsmI* knockouts. Thus, RsmE appears to either directly repress the production of these secretions or modulate the activity of other regulators that directly govern their production. The 5′-untranslated regions of the biosurfactant biosynthesis genes include a potential Rsm-binding site (44). However, it is difficult to predict genes that are directly regulated by Rsm proteins solely through a bioinformatics approach, since the Rsm-binding consensus sequence overlaps with the Shine-Dalgarno sequence (31–34). A recent study in Pseudomonas putida demonstrated that Rsm paralogs directly bind to both overlapping and unique mRNA, and RsmE appears to specifically regulate multiple regulators and extracellular products (30). In addition, Pseudomonas syringae possesses five Rsm paralogs, and three of them have been demonstrated to function in a nonoverlapping fashion to differentially regulate the production of diverse extracellular products and virulence genes (47).

We have shown that the production of both the mucoid polymer and the biosurfactant significantly boosts competition through spatial structure formation. The two key characteristics associated with the dominant spatial structure formed by the *rsmE* mutant are creation of space with a low cellular density and exclusion of the neighboring WT cells from this local environment (23). Here, we have demonstrated that the mucoid polymer is solely responsible for creating the space. We had initially interpreted our findings to indicate that the biosurfactant forms the exclusionary boundary, based on the mixed presence of the biosurfactant knockout and WT cells. However, we consistently observed that the WT cells rarely invaded deeply into the areas of low cellular density at high optical magnification. In addition, the borders of individual patches formed by the biosurfactant mutant were less defined and the mutant cells appeared to flow out on top of the neighboring WT cells, akin to lava flowing out from a volcano. However, these observations indirectly contradicted the results of our membrane assay, which showed that the same biosurfactant promoted the spreading of cells on the membrane surface. In fact, we initially referred to the corresponding secretion as a biosurfactant, due to the well-known function of bacterial surfactants in reducing surface tension to promote swarming on semisolid agar surfaces (48).

We identified the biosynthetic genes of the biosurfactant in this study, which were recently characterized by an independent group to produce a cyclic lipopeptide named gacamide A that promotes swarming (44). Pseudomonas spp. produce numerous cyclic lipopeptides that variably contribute to surface spreading and biofilm formation, and this variability potentially depends on discrete interactions with diverse extracellular or cell membrane-associated products (46, 49). The amphiphilic structure of gacamide A likely promotes its interaction with both hydrophilic compounds, like the mucoid polymer, and hydrophobic compounds that coaccumulate within the patches formed by the *rsmE* mutant. Importantly, removing the production of both the mucoid polymer and gacamide A maintained the respective *rsmE* mutant’s ability to outcompete the WT, albeit with much-reduced spatial dominance. These observations suggest that there are additional RsmE-regulated products that contribute to the competitive advantage of the *rsmE* mutant, which is clearly manifested through beneficial structures (23). Pressure likely builds up internally within a localized patch as the accumulating mucoid polymer constantly pushes away the surrounding WT cells to expand space. We thus speculate that gacamide A physically stabilizes the mucoid polymer and additional RsmE-regulated products to prevent their diffusion at the surface of the colony, which is uniquely devoid of neighboring cells and provides much less resistance.

A potential criticism of this study is the utilization of bacterial colonies to explore spatial structure formation, as these colonies lack important mechanical properties that manifest in natural microbial communities (7). However, resolving the problem of space and resource constraints in a densely populated colony likely shares common principles with other organisms in different experimental systems. Extracellular polysaccharides produced by Vibrio cholerae growing in microfluidic device biofilms promote the formation of isogenic structures that exclude the neighboring nonproducers (50), and glycolipid biosurfactants produced by Streptococcus spp. selectively displace competing genotypes on the tooth surface (51). In addition, cyclic lipopeptide production in Bacillus subtilis impacts the structure of fruiting body formation on an agar surface (52), but it is not essential (53). Our study also established a highly tractable experimental pipeline to identify and characterize additional RsmE-regulated products and to explore why RsmA and RsmI are functionally excluded from the formation of spatial structures.

## MATERIALS AND METHODS

### Strains and culture conditions.

Liquid and solid Lennox LB medium (Fisher) was used for general overnight cultures. Pseudomonas agar F (PAF; Difco) medium was used for all phenotypic screens, competitions, and microscopy. Pseudomonas minimum medium (PMM; 3.5 mM potassium phosphate dibasic trihydrate, 2.2 mM potassium phosphate monobasic, 0.8 mM ammonium sulfate, 100 mM magnesium sulfate, 100 mM sodium succinate) was used to selectively grow P. fluorescens isolates from conjugations with Escherichia coli donors. Routine cloning was carried out in E. coli 10B (Invitrogen) or E. coli JM109 (Promega), and E. coli S17.1λpir (54) was used as the donor strain in conjugations. When required, antibiotics were added to the medium at the following final concentrations: kanamycin (50 μg/mL), streptomycin (50 μg/mL), ampicillin (100 μg/mL), and gentamicin (20 μg/mL). P. fluorescens was cultured at 30°C or at room temperature (~22°C), and E. coli strains were cultured at 37°C. Liquid cultures were incubated with shaking at 250 rpm. All P. fluorescens strains used in this study are listed in Table 1.

**TABLE 1:** P. fluorescens strains used in this study

Strain	Relevant genotype	Relevant phenotype	Source
Pf0-1	WT	Nonmucoid, no biosurfactant	66
Pf0-1S	WT (Tn*7*-Sm^R^)	Streptomycin resistance	23
Pf0-1R	WT (Tn*7*-DsRed Express)	Red fluorescence	23
Δ*rsmE*	WT (Δ*Pfl01_1912*)	Mucoid, biosurfactant	23
Δ*rsmA*	WT (Δ*Pfl01_4273*)	Nonmucoid, no biosurfactant	This study
Δ*rsmI*	WT (Δ*Pfl01_4104*)	Nonmucoid, no biosurfactant	This study
M	WT (126th nucleotide deleted in *rsmE*)	Mucoid, biosurfactant	23
MK	M (Tn*7*-Km^R^)	Kanamycin resistance	23
MG	M (Tn*7*-Gfpmut2)	Green fluorescence	23
M*	M (Δ*Pfl01_3834*)	Nonmucoid, biosurfactant	42
M*K	M* (Tn*7*-Km^R^)	Kanamycin resistance	This study
M*G	M* (Tn*7*-Gfpmut2)	Green fluorescence	42
M^S^	M (*ΔPfl01_2211*)	Mucoid, no biosurfactant	This study
M^S^K	M^S^ (Tn*7*-Km^R^)	Kanamycin resistance	This study
M^S^G	M^S^ (Tn*7*-Gfpmut2)	Green fluorescence	This study
M^S^*	M (Δ*Pfl01_2211* Δ*Pfl01_3834*)	Nonmucoid, no biosurfactant	This study
M^S^*K	M^S^* (Tn*7*-Km^R^)	Kanamycin resistance	This study
M^S^*G	M^S^* (Tn*7*-Gfpmut2)	Green fluorescence	This study

### RT-qPCR.

Total RNA was isolated from colonies grown for 3 days at room temperature on PAF plates by using the TRIzol reagent (Thermo Fisher) following the manufacturer’s protocol. Total RNA quality and concentration were assessed using a NanoDrop spectrometer. First-strand cDNA synthesis was carried out using the High-Capacity RNA-to-cDNA kit (Applied Biosystems) with 1 μg of RNA and random hexamers, following the manufacturer’s protocol. Quantitative PCR (qPCR) optimized primers were obtained from Integrated DNA Technologies (see Table S1 in the supplemental material), and their quality was assessed through PCR with genomic DNA, cDNA, and no-reverse transcriptase (no-RT) cDNA reactions. qPCR was performed using SYBR green (Thermo Fisher) on the StepOnePlus instrument (Applied Biosystems). Each reaction was analyzed to ensure only one amplicon was amplified, by using dissociation curves. Gene expression was calculated using the 2^−ΔΔ^*^CT^* method with the 16S rRNA gene as an internal reference and quantified relative to *rsmI* expression (55).

### Biosurfactant assay.

Nuclepore Track-Etch polycarbonate membranes (Whatman; 0.4-μM pore size, 90-mm diameter) were used for assessing biosurfactant production. As previously described (23), one side of the membrane is shiny and the other is dull due to the manufacturing process. The dull side’s surface contains gaps and ridges that physically trap cells, but the biosurfactant permeates to produce a visible ring around colonies. The shiny side’s surface is smooth, which allows biosurfactant-producing cells to spread out through growth. Sterile forceps were used to overlay the membrane on the PAF agar surface, and 20 μL of overnight culture was spotted directly on the membrane and allowed to fully dry before the plates were inverted and incubated overnight at room temperature.

### Identification of biosurfactant biosynthesis genes by transposon mutagenesis.

Random transposon mutagenesis, using the plasmid pUT-mini*T*n5-Km*lacZ2* (56, 57) in E. coli S17.1λpir as the donor, was carried to identify the biosynthesis genes of the biosurfactant, as previously described to identify the biosynthesis genes of the mucoid polymer (53). Briefly, overnight cultures of the donor and M* strains were washed in PMM, mixed at the relative ratio of 1:6, spotted on solid LB to conjugate, incubated at 30°C for 3 h, harvested, and plated out on solid PMM supplemented with kanamycin. Over 20,000 transconjugant colonies were picked and rearrayed using the QBot system (Genetix) into 384-well plates containing kanamycin-supplemented PMM and then incubated at 30°C. Surfactant assays on overnight cultures were conducted on PMM plates overlaid with the dull side of the polycarbonate membrane, as described above, with a disposable 384-pin replicator (Scinomix). Mutants that were defective in biosurfactant production (dull side) were rearrayed into 96-well plates containing kanamycin-supplemented PMM and then incubated at 30°C. Overnight cultures were retested for biosurfactant production as described above using a disposable 96-pin replicator (Scinomix). Mutants that failed to produce the biosurfactant ring were selected, ignoring those that had obvious growth defects. The transposon insertion sites were identified by arbitrary primed PCR as previously described (53).

### Mutant construction, complementation, and tagging.

Gene deletion mutants were constructed by the gene splicing by overlap extension method (58), using the plasmid pMQ30 (59) or pSR47s (60) as previously outlined (23, 42). PCR primers used to construct and confirm each mutation are listed in Table S2. Briefly, for each targeted gene, approximately 500 bp of its flanking upstream and downstream regions were individually amplified, joined together, first cloned into the pGEM-T Easy vector system (Promega), then subcloned into pMQ30 or pSR47s, and transformed into E. coli S17.1λpir as the donor strain. Overnight cultures of the donor and target strains were washed in PMM and mixed at an equal ratio, spotted on solid LB, incubated at 30°C overnight, harvested, and plated out on solid PMM supplemented with gentamicin (pMQ30) or kanamycin (pSR47s). Transformants were grown on solid LB supplemented with sucrose (5% [wt/vol]) overnight, and the resulting colonies were screened using primers that bind outside the two flanking fragments for the expected reduction in amplicon size. To confirm the gene deletions, we isolated genomic DNA from overnight cultures using the DNeasy UltraClean microbial kit (Qiagen) following the manufacturer’s protocol, and whole-genome sequencing was conducted at the Microbial Genome Sequencing Center (MiGS; Pittsburgh, PA). Kanamycin-resistant and streptomycin-resistant strains used in competitions and GFP-tagged and DsRed Express-tagged strains used in microscopy were constructed using the mini-Tn*7* chromosomal insertion system (61) as previously described (23, 42). The same set of the kanamycin and streptomycin resistance markers utilized in this study has been demonstrated to be neutral for conducting competition experiments in P. fluorescens Pf0-1 (23). The Δ*rsmE* mutant was complemented by integrating the native *rsmE* locus into its chromosome by using the mini-Tn*7* system (pHRB2) via conjugation as previously described (62). The native *rsmE locus*, including the 500-bp upstream and 500-bp downstream sequences from the open reading frame, was PCR amplified from P. fluorescens Pf0-1 using primers RsmE1 (5′-CGCTGGCATCCTTGATGACG) and RsmE2 (5′-TCTGGATCCGGTGAGGTCGC). The amplified product was cloned into pGEMT-Easy, then subcloned into pHRB2 using the ApaI and EcoRI sites, and introduced into the Δ*rsmE* mutant as described above. Chromosomal integration of the native *rsmE* locus into the noncoding Tn*7* insertion site was confirmed by whole-genome sequencing.

### Measurement of monoculture growth.

For the measurement of growth in colonies, overnight cultures were resuspended in PMM and 20 μL was spotted on PAF plates and incubated at room temperature. To enumerate the initial population size, each cell suspension in PMM was serially diluted and plated on LB plates, and resulting colonies were counted on the following day. Three spotted colonies were scraped on each day over 7 days and resuspended in 5 mL of PMM using a sterilized bent glass Pasteur pipette. Cell suspensions were vortexed until clumps were no longer visible and then serially diluted and enumerated as described above. For the measurement of growth in liquid, overnight cultures were diluted into a manually formulated PAF without agar (63) in six replicates, and optical density at 600 nm was measured every 30 min over 48 h (30°C, constant shaking) in the Bioscreen C MBR system (Oy Growth Curves Ab Ltd.).

### Competition assay.

Competitions between kanamycin-resistant mutant strains and the streptomycin-resistant WT strain were conducted as previously described (23). Briefly, overnight cultures (1.5 mL) were washed in fresh PMM and resuspended in 1.0 mL PMM, and the mutant strain suspension was serially diluted to 10^−3^ in PMM and mixed with equal volumes of the undiluted WT strain suspension. Twenty microliters of each competition mixture was spotted in triplicate on a PAF plate and incubated at room temperature. To enumerate the initial population sizes of the competing strains, each competition mixture was serially diluted and plated on LB plates supplemented with either kanamycin or streptomycin, and resulting colonies were counted on the following day. Four or seven days later, the spotted colonies were scraped and resuspended in 5 mL of PMM, serially diluted, plated, and counted as for the initial competition mixture. The results of the competitions were analyzed by calculating the relative fitness (*W*) of each competing strain against the WT (64), as follows: [ln(CFU of mutant at time of sampling/CFU of mutant at time zero)]/[ln(CFU of WT at time of sampling/CFU of WT at time zero)].

### Statistical analysis.

Competition experiments were conducted with at least three biological replicates and two technical replicates for each biological replicate. The data were first analyzed with an analysis of variance (ANOVA) to evaluate if the means of the biological replicates differed significantly, and then Tukey’s honest significant difference test (*P* < 0.05) was applied to make multiple pairwise comparisons within the data set. All comparisons were found to be statistically different or are noted as nonsignificant. Statistical tests were conducted using GraphPad Prism.

### Microscopy.

Overnight cultures of GFP-labeled strains and DsRed Express-labeled WT were washed and resuspended in PMM. All GFP-labeled cell suspensions were serially diluted to 10^−5^ in PMM and mixed with equal volumes of the undiluted DsRed Express-labeled WT suspension. Twenty microliters of each competition mixture was spotted in triplicate on PAF plates and incubated at room temperature. Epifluorescence microscopy was conducted using the Nikon SMZ25 stereo compound microscope with 0.5× and 2× SHR Plan Apo objectives and the NIS Elements software. For confocal microscopy, an agar slice containing the entire colony was placed on a microscope slide and visualized without a coverslip. Confocal microscopy was conducted using a Nikon Ti2 microscope with the air-corrected 100× TU Plan Apo objective and the NIS Elements software. Nonfluorescent imaging of colonies was carried out using the Hayear overhead microscope (HY-2307) or the Canon Rebel EOS T3 DSLR camera. Images were rendered using the NIS Elements and ImageJ software.

### Data availability.

All noncommercial plasmids or strains used in this study are available for distribution upon request.
