# Supplementary material for: Spatial Structure Formation by RsmE-Regulated Extracellular Secretions in Pseudomonas fluorescens Pf0-1
Source: J Bacteriol. 2022 Sep 27;204(10):e00285-22. doi: 10.1128/jb.00285-22 (PMC9578434; doi:10.1128/jb.00285-22)
Supplement: Supplemental file 1 — Fig. S1 to S4 and Table S1. Download jb.00285-22-s0001.pdf, PDF file, 0.5 MB [file jb.00285-22-s0001.pdf]

Spatial structure formation by RsmE-regulated extracellular secretions in *Pseudomonas fluorescens* Pf0-1

Anton Evans, Meghan Wells, Jordan Denk, William Mazza, Raziel Santos, Amber Delprince, and Wook Kim*

Department of Biological Sciences, Duquesne University, Pittsburgh PA, 15282

*Correspondence to Wook Kim: kimw1@duq.edu

Supplemental Material

Figures S1-S4 and Table S1

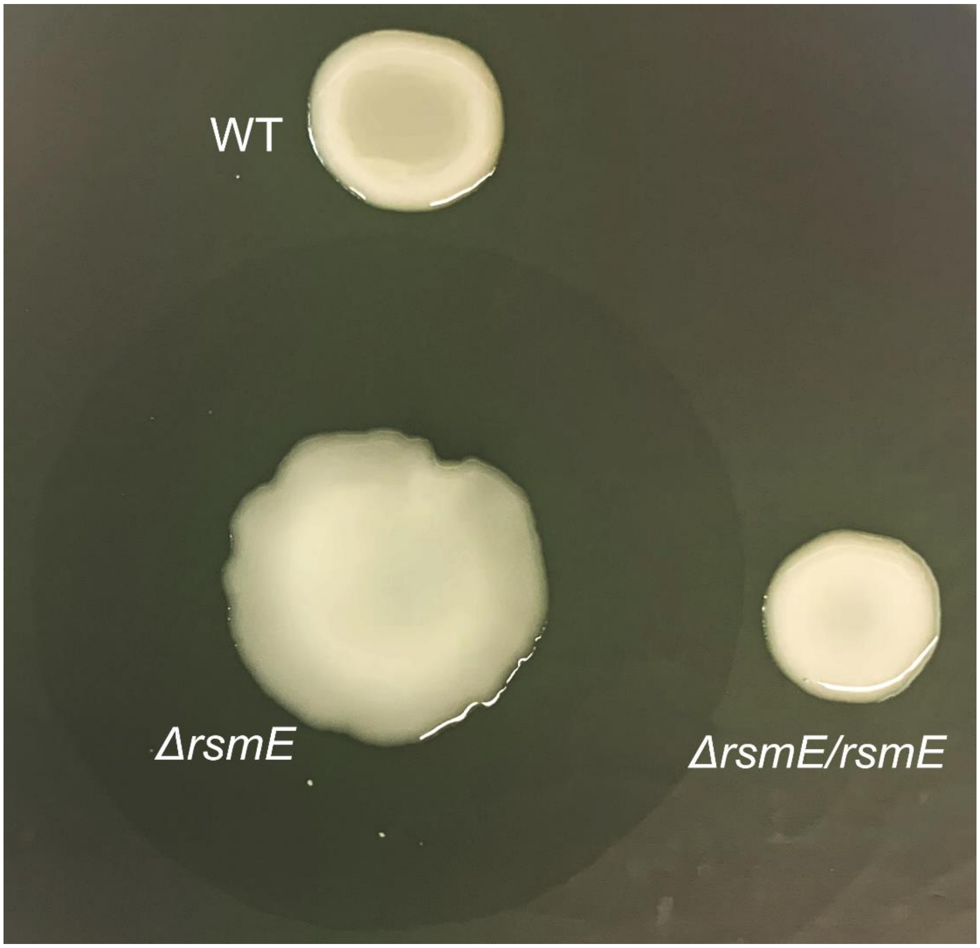

Figure S1. Genetic complementation of the ∆*rsmE* strain with the native *rsmE* locus from the WT restores the WT phenotype. The ∆*rsmE* strain produces the biosurfactant ring on the dull side of the polycarbonate membrane, but the WT does not. Chromosomal insertion of the native *rsmE* locus in the ∆*rsmE* mutant restores the phenotype exhibited by WT. The image was captured after 3 days of incubation.

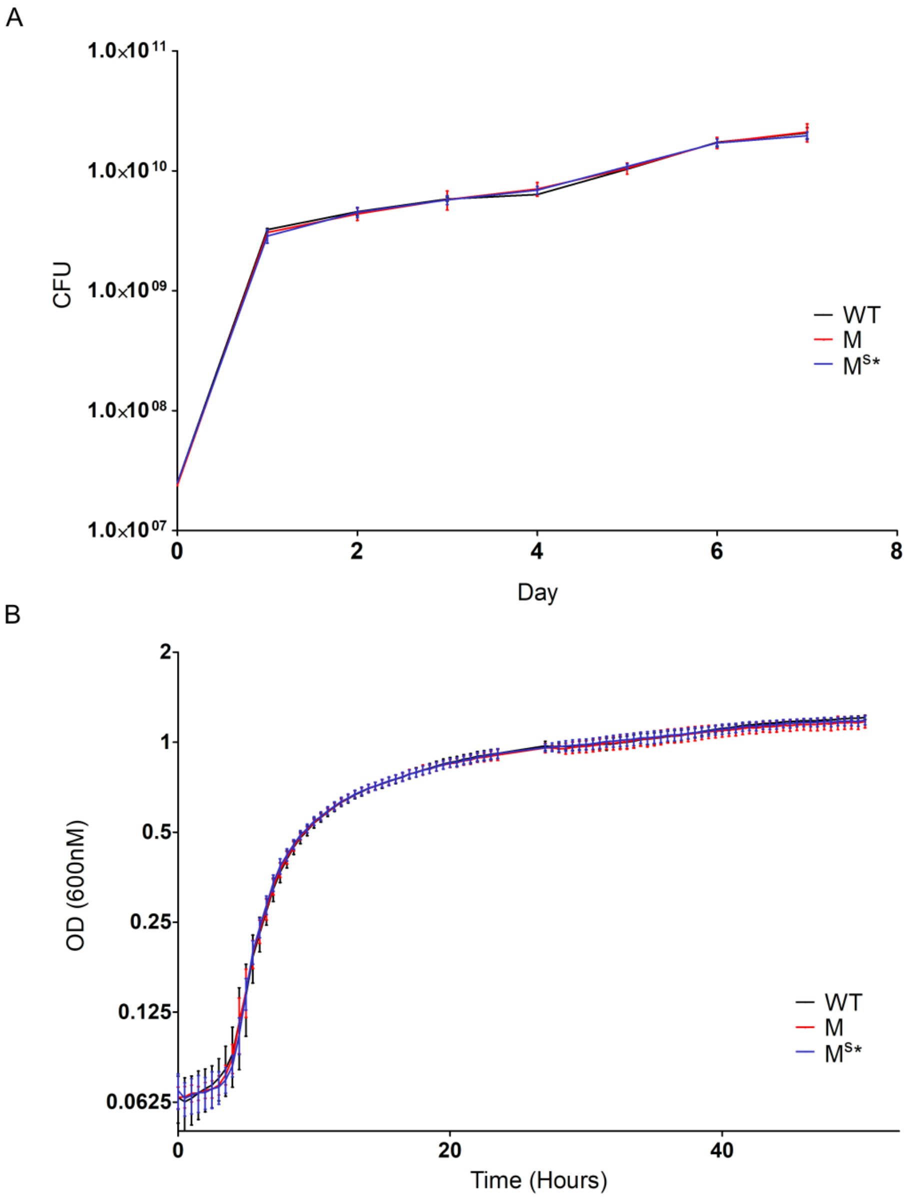

Figure S2. Production of RsmE-regulated extracellular secretions does not impact growth in monoculture. (A) Growth profiles of single genotype colonies of WT, M, and M^{S*} on solid PAF. Each data point represents the mean colony forming units (CFU) of three populations and the error bars represent the standard deviation of the mean. (B) Growth profiles of single genotypes in liquid PAF as measured by optical density at 600 nm. Shown are the mean of six independent cultures for each strain, and the error bars represent the 95% confidence interval.

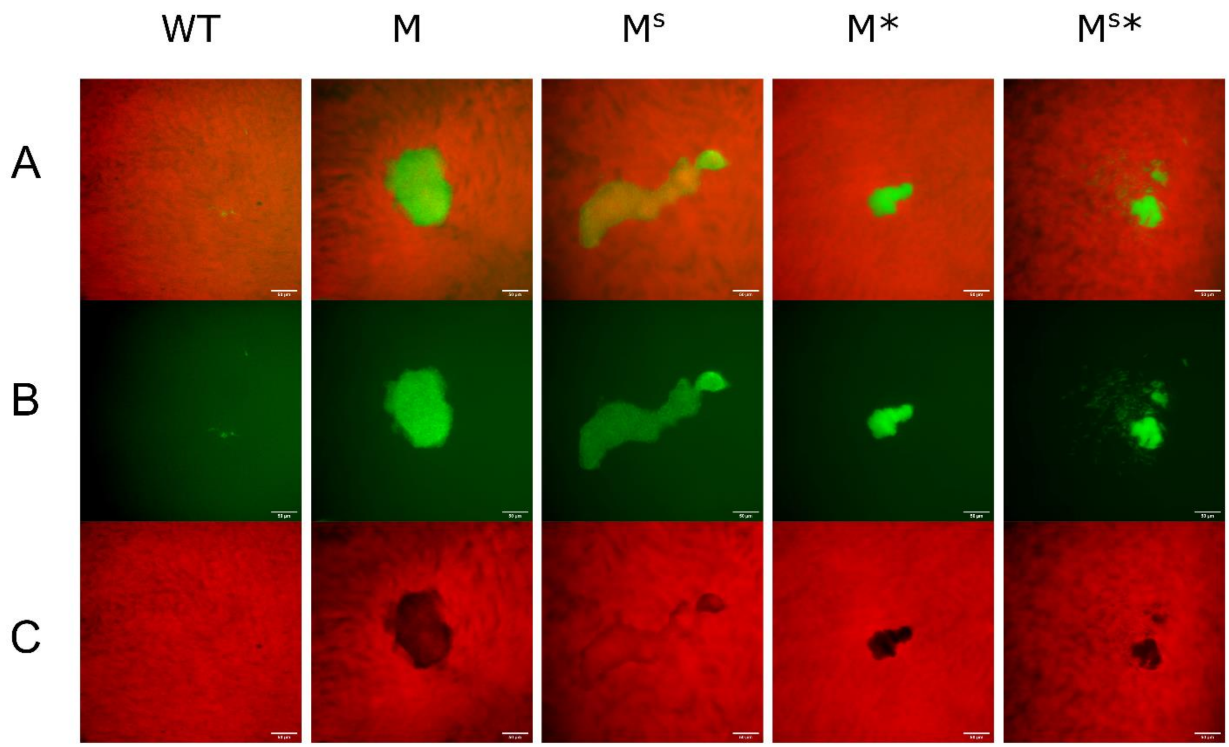

Figure S3. Green and red channel epifluorescence microscopy images from Fig. 5B. Each indicated strain was chromosomally tagged with GFP, heavily under-represented in a mixture with DsRed-Express-tagged WT, and representative co-cultured colonies were imaged five days later. (A) Same images as shown in Fig 5B, in which the green and red channels images are overlaid. (B) Green channel images showing only GFP-expressing cells. (C) Red channel images showing only WT cells expressing DsRed-Express. Scale bar represents 50 um.

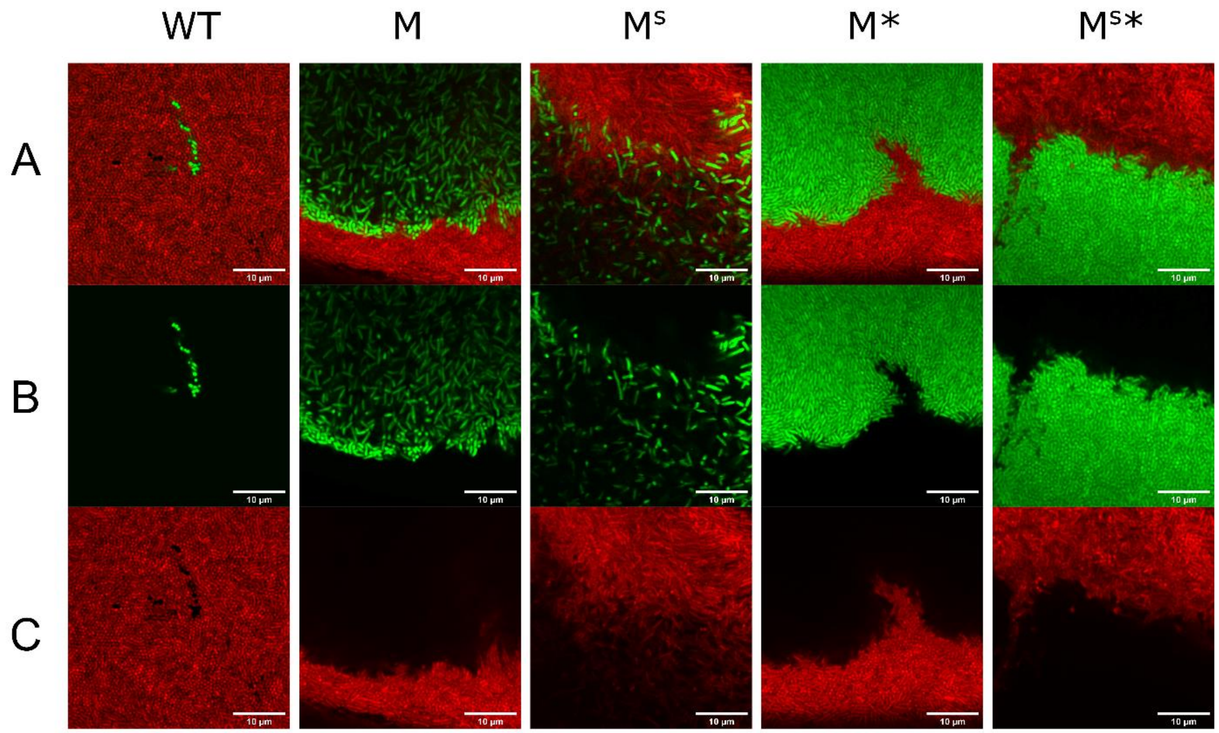

Figure S4. Green and red channel confocal microscopy images from Fig. 5C. Each indicated strain was chromosomally tagged with GFP, heavily under-represented in a mixture with DsRed-Express-tagged WT, and representative co-cultured colonies were imaged five days later. (A) Same images as shown in Fig 5C, in which the green and red channel images are overlaid. (B) Green channel image showing only GFP-expressing cells. (C) Red channel images showing only WT cells expressing DsRed-Express. Scale bar represents 10 um.

Table S1. Primers used in this study.

Name	Function[a]	Sequence
M^S-1	Outside F	CCCAACTGCCAATAAAAGTGCGG
M^S-2	Outside R	TGGGCAAATGCATCGAGGCG
M^S-3	Up F	TCCATGTTGCGAAGACTGCCG
M^S-4	Up R	CCCGTGACCGATCAGTCATGGAAGATCCATCTCACGTGATTTGGCG
M^S-5	Down F	CGCCAAATCACGTGAGATGGATCTTCCATGACTGATCGGTCACGGG
M^S-6	Down R	CAGATCGCCTTCGCCAGCCG
M*-1	Outside F	TGACGATGGCCATGTGTTGCAGG
M*-2	Outside R	GCCGGGGGAGAACATGCAAC
M*-3	Up F	CGTCCCGTGCTTCTTCAACG
M*-4	Up R	CTAACATTGCACTAAAACGCCATCAAGTGGGAA
M*-5	Down F	CGAGTTGGCACTTTTCCCACTTGATGGCGTTTTA
M*-6	Down R	TCCAAAAACAAAGTCACCCG
rsmA-1	Outside F	GTGTCTACACCACTGACCCG
rsmA-2	Outside R	CAGAAAGCGAGAAATGGCCG
rsmA-3	Up F	CTACCAGTCCGCGCAAACC
rsmA-4	Up R	TCTTCCCCGTTTGCAAACATACCTTTCTCCTCACGCGAATC
rsmA-5	Down F	GATTCGCGTGAGGAGAAAGGTATGTTTGCAAACGGGGAAGA
rsmA-6	Down R	GGATTCGAACCTGTGACCGC
rsmI-1	Outside F	GCAACAGTCACCCTGACG
rsmI-2	Outside R	GAGCGCACGCACATCGAC
rsmI-3	Up F	GATGTCTTGCATGCTCAATTACC
rsmI-4	Up R	CCTGTTCGACTGAAAACGGGGGAGGTGTCCTTCAAGG
rsmI-5	Down F	CCTTGAAGGACACCTCCCCCGTTTTCAGTCGAACAGG
rsmI-6	Down R	CTGGCGGCGCTCAGTCG
Pfl01_F	*16S* qPCR F	GCGTAGGTGGTTCGTTAAGT
Pfl01_R	*16S* qPCR R	CACCACCCTCTACCATACTCTA
*rsmE*_F	*rsmE* qPCR F	TGACATCACGATCACCATTCTC
*rsmE*_R	*rsmE* qPCR R	GTAGATCTCTTCCCGGTGTACT
*rsmA*_F	*rsmA* qPCR F	TGCTCGGCGTTAAAGGAAA
*rsmA*_R	*rsmA* qPCR R	CGCAGATAGATTTCTTCACGGT
*rsmI*_F	*rsmI* qPCR F	CATCACTCTGCGCGTTCT
*rsmI*_R	*rsmI* qPCR R	TTTGCGCTGGATCCGTT

[a] For each gene target, Outside F/Outside R primers were used to screen for deletions, Up F/Up R primers were used to amplify the upstream fragment, and Down F/ Down R primers were used to amplify the downstream fragment. qPCR F/R primers were used to conduct qPCR of the indicated gene.
